# The impact of Covid-19 on professional identity formation: an international qualitative study of medical students’ reflective entries in a Global Creative Competition

**DOI:** 10.1186/s12909-022-03595-1

**Published:** 2022-07-15

**Authors:** Zoe Moula, Jo Horsburgh, Katie Scott, Tom Rozier-Hope, Sonia Kumar

**Affiliations:** Medical Education Innovation & Research Centre, Imperial College London, London, UK

**Keywords:** Professional identity formation, Disorienting dilemmas, Arts and humanities, Transformative learning

## Abstract

**Introduction:**

The Covid-19 pandemic, which affected medical students globally, could be viewed as a disorientating dilemma with the potential to offer opportunities for transformative learning. In 2021 the Medical Education Innovation and Research Centre at Imperial College London launched a Global Creative Competition as a platform for medical students to reflect on their experiences during the pandemic.

**Methods:**

Six hundred forty-eight creative pieces with written reflections were submitted by medical students from 52 countries. 155 students from 28 countries consented for their entries to be included in this study. The reflections were analysed thematically and independently by three reviewers to explore how the pandemic impacted students’ professional identity formation (PIF).

**Results:**

The pandemic increased students’ awareness of the social and global role of doctors in addressing health inequities. Students felt part of a wider healthcare community and showed greater appreciation towards person-centred care. Students also became more aware of their personal needs, priorities, and the importance of self-care.

**Discussion:**

In agreement with Mezirow’s theory of transformative learning (2003), the pandemic led students to re-examine pre-existing epistemic and sociocultural assumptions concerning the role of doctors and explore new perspectives of what it means to be a doctor. In accordance with Cheng’s theory of coping flexibility (2021), students developed both emotion-focused coping strategies (e.g., arts engagement) and problem-solving strategies (e.g., volunteering), suggesting they were able to adjust psychologically and develop agency. However, students experienced tension between their sense of duty and sense of wellbeing, highlighting the need for medical educators to design into programmes formal support systems where medical students have the space and time they need to reflect on their emergent identities as a doctor.

**Conclusion:**

Medical educators should encourage students to reflect on their identity formation while encountering disorientating dilemmas. The inclusion of arts and humanities within the medical curriculum is strongly recommended to provide an avenue for students to access and express complex emotions and experiences.

## Introduction

The Covid-19 pandemic has affected medical education globally through the closure of university campuses, transition to online learning, interruptions in clinical training, and increasing pressures on health systems. With such sudden and unpredicted changes, medical students were often ill prepared for the challenges they were suddenly exposed to, such as end-of-life experiences, witnessing traumatic clinical situations, long shifts, health risks from a lack of adequate personal protective equipment (PPE), as well as conflicts with moral and ethical duties [1]. Such lack of ‘pandemic preparedness’ [1] was seen with medical students in a variety of contexts; a survey with 273 medical students across all years at the National University of Ireland found that only 4% felt confident that they were prepared to work in the frontline, while only 23% felt their skills would be useful in emergency situations [2]. Another survey found that the most common problems that students experienced due to Covid-19 were related to their productivity (27%), physical health (26%), and emotional health (14%) [3]. Significantly higher levels of severe stress were reported during the pandemic from medical students in the later clinical years due in terms of students’ learning needs, teaching patterns and clinical preparedness [4].

Professional Identity Formation (PIF) has been recognised internationally as a central aim of medical education [5] and has been defined as the ability to ‘think, act, and feel like a physician’ [6]. It involves the process by which medical students gradually adopt the values, dispositions, and aspirations of the physician community [7]. Considering that PIF has wider implications for medical students’ health, wellbeing and job satisfaction [8], investigating the impact of Covid-19 on medical students’ PIF from a global perspective may indicate actions that medical schools need to take in the post-pandemic era.

The Covid 19 pandemic has been considered to be a disorientating dilemma [9], a concept central to Mezirow’s theory of transformative learning [10]. These dilemmas allow us to engage in a process of transformation, as our experiences challenge our thinking and existing meaning structures. Experiences of disorienting dilemmas, such as the Covid-19 pandemic, can provide opportunities for critical reflection and assessment of pre-existing epistemic, sociocultural, or psychological assumptions, developing the basis of transformative learning. This struggle with unquestioned assumptions allows learners to explore alternative ways of being and living in their relationships, roles and actions, as well as to employ new, transformed perspectives [9]. The uncertainty and ambiguity caused by disorienting dilemmas are a central component of PIF as they result in tensions between personal values and professional duties [11]. Although, there is growing research exploring how the pandemic affected students’ PIF, most studies are undertaken in individual universities or countries. This study offers a unique insight from an international perspective in the use of humanities to understand how global issues such as the pandemic can impact on learner experiences.

### Global creative competition

In August 2020, the Medical Education Innovation and Research Centre (MEdIC) at Imperial College London launched a Global Creative Competition. The aim of this was to provide a platform for medical students around the world to reflect on their personal and professional experiences during the pandemic and produce a creative response to this. Medical students were invited to submit their creative submissions under two categories: a) visual submissions (e.g., paintings, drawings, comics, photographs) or b) literary submissions (e.g., poems, stories, song lyrics). They were also asked to submit up to 250-word reflection explanation of their submission (in English), outlining why they chose it, what it meant to them personally and in their role as a future doctor.

The General Medicine Council [12] has suggested that arts-based reflective practice can foster wellbeing, resilience, self-awareness, and combat potential student burnout. This has been confirmed by a growing body of literature. For example, a study exploring the impact of comic-making on medical students’ PIF found that this process enabled students to reflect on transformative experiences, connect better with their patients, identify the triumphs and challenges of being a doctor, develop clinical reasoning and attention to nonverbal cues [13]. Stephens et al. study [14] found that mask-making supported medical students to explore the impact of sociocultural changes on their PIF. Cox et al. [15] found that artmaking allowed medical students to discover their professional identity, and this contributed towards an improved sense of wellbeing and commitment towards a better future for medicine. In several other studies, engagement with the arts facilitated emotional and self-expression [14–16] enabled medical students to realise their interpersonal, perceptual, and expressive capacities [17], offering a sense of personal growth and empowerment [18].

Based on the existing literature, an analysis of students’ reflections triggered by their creative responses may provide unique insights regarding the impact of the pandemic on students’ PIF, whether their understanding of the role of doctor changed in light of Covid-19, and how medical students navigated these changes. Therefore, our research question was:

- How has the Covid-19 pandemic impacted on medical students’ PIF?
  ◦ how students’ perceptions of the role of the doctor have changed?
  ◦ how students’ perceptions of their own role as a future doctor have changed?
  ◦ how students navigated these changes?

## Methodology

### Research approach

We considered PIF to be a fluid and dynamic process [19] and as such, students’ written reflections would provide useful insights into how they viewed their professional identity and navigated any changes in their role as a future doctor during a global pandemic. Given the international nature of the Global Creative Competition, we also considered this an opportunity to better understand the experience of medical students from across the world. The application guidelines and website information are available through this link: Global Creative Competition: Medical Student Responses to COVID-19 | Faculty of Medicine | Imperial College London.

### Participants

**Fig. 1:**
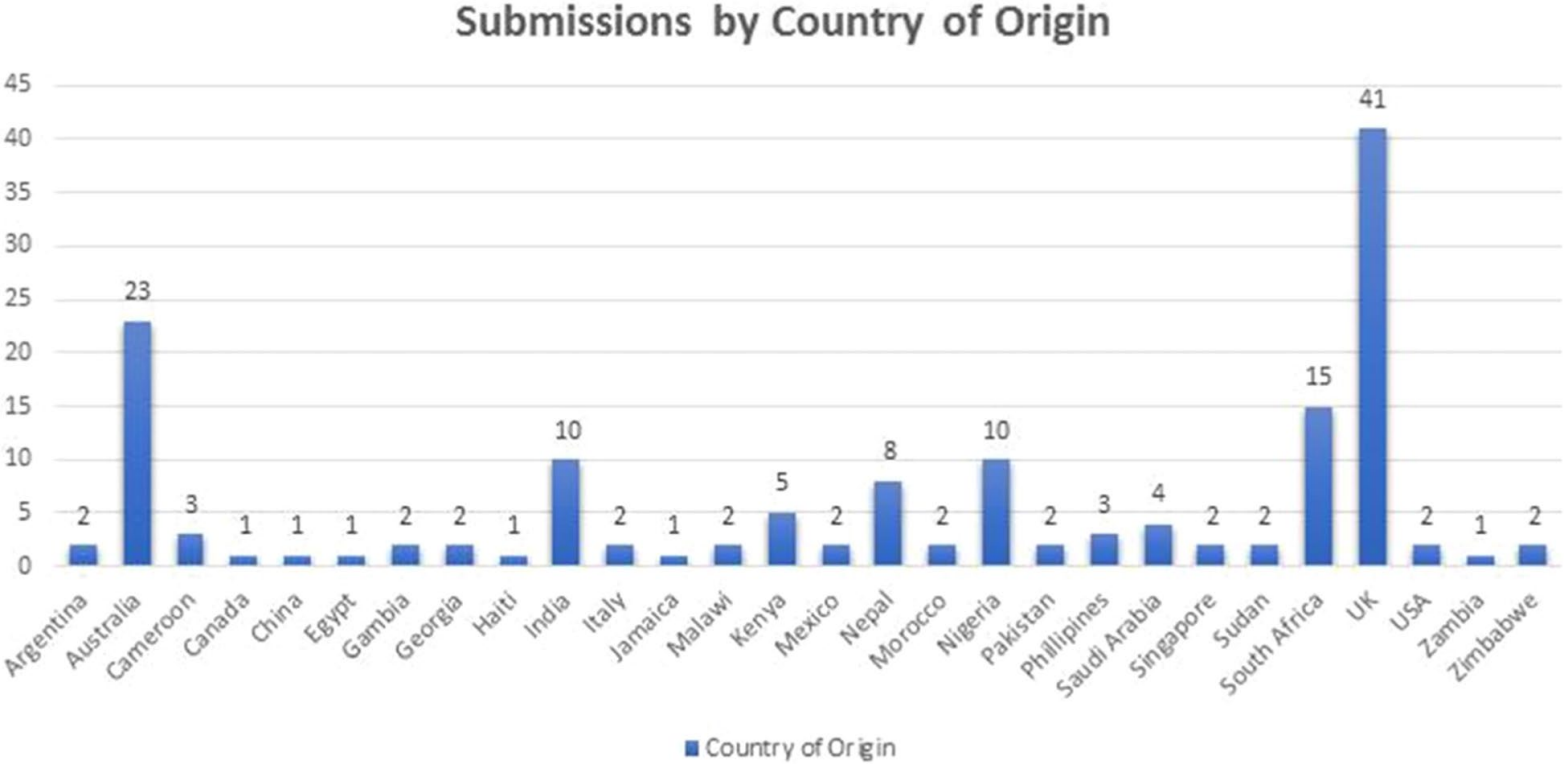
Submissions by country of origin

Medical students from across the world were eligible to submit their creative pieces with written reflections to the competition. We received submissions from 648 medical students from 52 countries. Following the competition all students were emailed and invited to have their reflection included in the data set for this study. Out of 648 students, 155 from 28 countries consented for their reflection to be included in our analysis. The data we collected was related to students’ medical school, academic year, and country of origin (Fig. 1), which were anonymised post-collection by ZM. However, we did not collect any personal data, such as students’ age, gender, ethnicity, or why they did not consent to participate in the research as this was an optional part of the Global Creative Competition.

### Data collection and analysis

Students submitted their visual or literary creative piece alongside a 250-word reflection in which they were asked to outline why they chose it, what it meant to them personally and in their role as a future doctor. These reflections where then thematically analysed, and an iterative approach was taken to this analysis. Three researchers (ZM, JH, KS) conducted a pilot phase with 20 reflections (five different, five in common), to support the development of the coding framework. This was then shared with the whole research team and further refinement was made to the codes until consensus was reached [20]. Each of these authors (ZM, JH, KS, TD) then undertook analysis of a set of reflections using the agreed upon coding framework. Each of these authors also wrote a short research ‘memo’ outlining the key themes and initial reflections on the data. This analysis and memos were then discussed with the wider authorship team and summaries were produced for each theme. An ontologically constructivist and epistemologically interpretivist assumption was adopted, and as such, we acknowledge the existence of multiple subjective realities which are socially constructed by each student individually [21]. As a research team, we made an effort to understand these individual realities, yet we also recognise that students’ reflections can be interpreted from various perspectives, depending on our own background, positionality, and areas of expertise. For this reason, it was important to develop a code book that was agreed by the whole team, and to have frequent discussions regarding the interpretation of the results at all stages.

### Ethical issues

The study was granted ethical approval by Imperial College London Education Ethics Review Process (Ref no.: EERP2021-030). Informed consent was sought from participants, and they were able to withdraw from the study at any time up to the point of data anonymisation. A coding system (i.e., data labelled with pseudonyms) was always in place to ensure that data anonymity and confidentiality was protected. Since all data were electronic, they were stored and backed up at Imperial College London devices, which were password protected.

## Results

We analysed a dataset of 155 reflective entries. The pandemic appeared to impact medical students’ PIF primarily in five areas:

- a) Awareness of social accountability.
- b) Awareness of global role of the doctor in addressing health inequities.
- c) Becoming a member of the wider healthcare community.
- e) Appreciation of person-centred care.
- f) Awareness of personal needs and self-care.

Mezirow's theory [10] consists of ten stages, the first being the disorientating dilemma and the Covid-19 pandemic has been argued to be such an experience. The second stage of this theory is about self-examination, and we argue that engagement with the production of a piece of art and its reflection involves self-examination of the students' experience and feelings in relation to the disorientating dilemma. Many of the reflections then addressed subsequent stages of Mezirow's theory. For example, step three involves assessment of existing assumptions about the world which can be seen where students reflected on their greater awareness about the role of the doctor, exacerbated health inequalities due to Covid-19, and their role as a medical student and member of the healthcare community.

### Awareness of social accountability

Students reflected on their social responsibility to educate their communities about Covid-19, such as raising awareness regarding symptoms and how spreading the infection could be prevented. The students also expressed their desire to contribute to other local efforts through charity fundraising, volunteering in healthcare settings, or through more simple methods such as role modelling mask wearing. Such activities provided them with a sense of purpose and a feeling of being useful. For many students the pandemic also highlighted the limited access to medical provision for their local communities. This motivated students to advocate for vulnerable patients and to appreciate the value of being a socially accountable doctor.

“I started volunteering at a care home […] I was horrified at the discrimination that individuals faced when trying to access healthcare, refused care or resources such as chemotherapy and surgery. It was in this care home that I met Chris – a man diagnosed with Down syndrome and dementia. Chris contracted COVID and was refused treatment at most public hospitals – he passed away. This piece [the creative response submitted] focuses on the hopelessness I felt as a medical student trying to save someone who society had sentenced to death. Chris will inspire me to continue to be the best doctor I can possibly be.” – 3rd year, Pretoria, South Africa.

### Awareness of global role of the doctor in addressing health inequities

Students referred to the pandemic highlighting significant inequality both in their own countries and across the world. They commented on socioeconomic and health disparities, unemployment, and homelessness, with some highlighting struggles in their own countries on issues such as government corruption and poor infrastructure. The experience and recognition of these injustices and inequities served as a point of inspiration to the students in their future role as a doctor, and that addressing some of these challenges, such as advocating against racism and discrimination, would form the kind of doctor they would like to become.

“This pandemic has further exacerbated wealth, racial and health inequalities. As long as we refuse to actively address racism in medicine and medical education, we cannot be good doctors. We must recognise our biases and call others out on theirs. Silence is complicity. As a Black woman, I want to work in a system that would advocate for me; as a doctor and a patient” – 3rd year, Queensland, Australia.

**Fig. 2:**
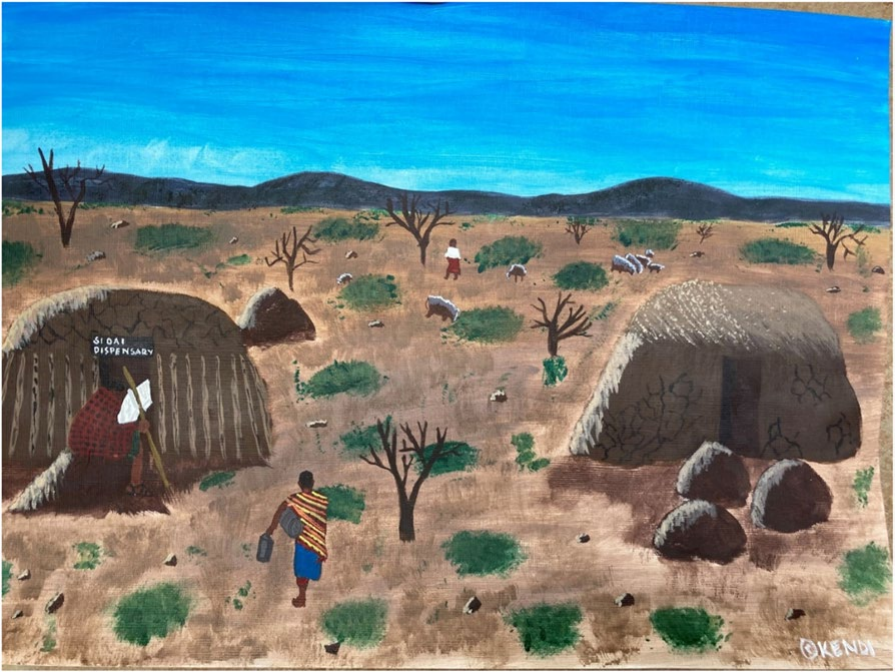
Awareness of global role of the doctor in addressing health inequities

“I chose this painting because my mind has really been buzzing thinking about marginalized communities in my country, wondering how they’re coping. I would like to find ways of having much more accessible health care to people in rural areas, where the standards of care will be better, affordable and around the clock” – 3rd year, Nairobi, Kenya (Fig. 2).

### Becoming a member of the wider healthcare community

Students reflected upon the importance of working collectively in multi-disciplinary teams, including healthcare professionals, charities, and governmental organisations.

**Fig. 3:**
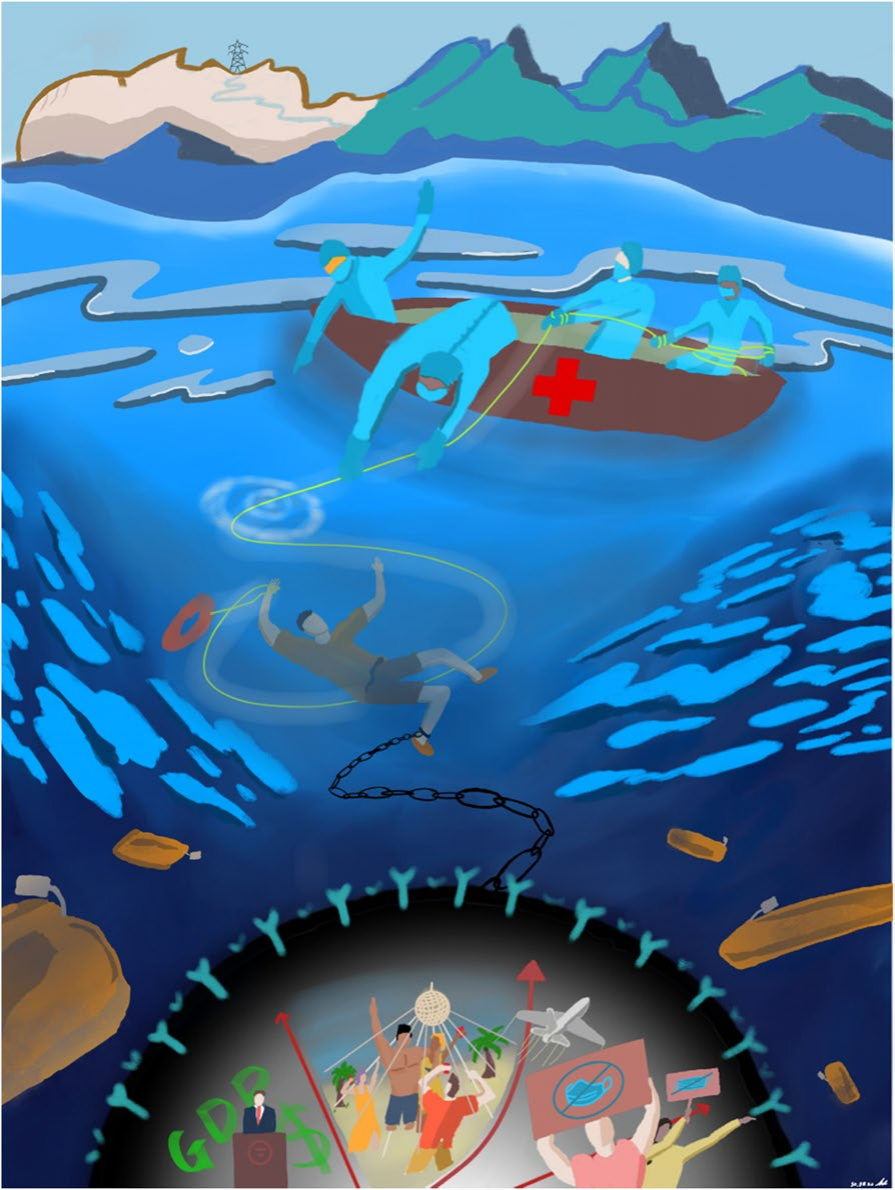
Becoming a member of the wider healthcare community

“My illustration reflects the heroic efforts of healthcare workers in bringing patients back from the brinks of death. This is reflected as a person drowning to the ocean depths while chained to a prison ball, while healthcare workers try to pull him out of the water. There is emphasis on teamwork and multi-disciplinary teams, which is paramount in our future practice in medicine.” – 1st year, Sydney, Australia (Fig. 3).

Medical students expressed a sense of pride at being part of a healthcare community that makes a great impact on people’s lives, even in the circumstances where students had to deprioritise their own needs for the benefits of others:

“These students, who a month back were partying, studying and having a great time, were suddenly required to get into PPE suits and in literal sense " save the world". This makes me proud of the medical fraternity and fortunate to be a part of it” – 3rd year, Jhansi, India.

Some students also suggested that the pandemic was a call for greater unity beyond the local or national level, and an opportunity for a truly global medicine:

“Although medical students around the world are separated by thousands of kilometres of land, we are all connected by the same energy, the same goal of being part of the medical community and helping as many people we can” – 6th year, San Luis Potosi, Mexico.

### Appreciation of person-centred care

Students expressed that the experiences of the pandemic allowed them to recognise more than ever the importance of person-centred approaches, such as active listening, empathy, and understanding of the patients’ needs. They expressed their motivation to teach and adopt person-centred care in their role as future doctors. As students mentioned:

“I’d like to advocate for mental health and teaching people the empathetic dialogue of identifying when someone is not O.K. As a future doctor I aim to make the necessary changes personally and systematically so that patients feel cared” – 1st year, Queensland, Australia.

“It has made me realise that when I qualify, I hope that I am able to show my patients that I really care about helping them to the best of my ability and to ensure that they feel supported and treated as individuals. I hope to care for my patients in the future in the way that I would like to be treated if I was a patient” – 4th year, Buckingham, UK.

### Awareness of personal needs and self-care

The pandemic inevitably had a strong impact on medical students’ mental health and wellbeing. Students expressed feelings of loneliness, helplessness, frustration, anxiety and depression. Some students also witnessed doctors making personal sacrifices to serve their communities, such as putting themselves in danger, being overworked and stressed, and prioritising the wellbeing of others ahead of themselves. For some students, not unexpectedly, these observations caused distress, for example:

“I think to myself that one day, I will get to help people like my senior colleagues and my heart swells with joy. But when I see how overworked doctors are, how stressed they look, and not to mention how exhausting medical school is, I can’t help but feel a little discouraged.” – 4th year, Lagos, Nigeria.

Such observations triggered students to develop self-awareness, understand and prioritise their own health needs. For example, some students became increasingly aware that success, recognition, and validation might not bring life satisfaction. As a result, these markers of external validation became less important, for example:

For the first time, I saw my life and pursuits clearly—chasing after success, recognition, validation, and all that I thought would satisfy but never did.” – 1st year, Manchester, UK.

**Fig. 4:**
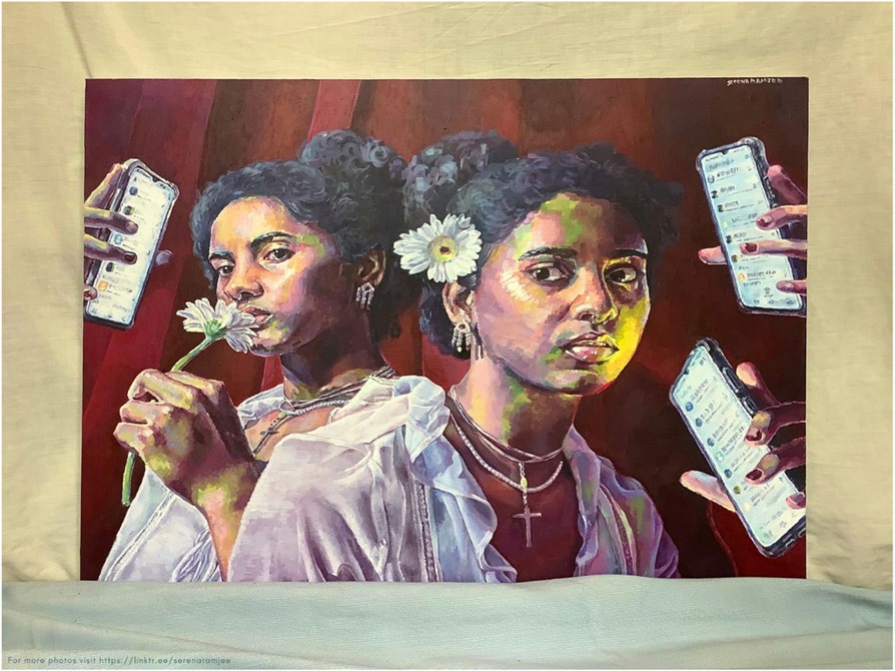
Awareness of personal needs and self-care

“"_missed calls" aims to capture this pressure and the feeling of only having myself for company. My isolation accentuated my other symptoms and propelled me into a depressive state. Recovery began with prioritising my health before work and visiting my GP. My experience during these past months has made me examine my behaviour before lockdown and acknowledge my toxic productivity. I contemplated whether my deterioration was inevitable due to my inability to relax and how this could affect me in the future” – 3rd year, London, UK (Fig. 4).

Some students also mentioned that they discovered new coping mechanisms, such as mindful walking, engaging with the arts, and appreciation of life and nature.

“I took this photo during week 8 of lockdown. I was bored and lacked motivation. I decided to pick up my camera and photograph random things that I would often overlook. I decided to look up at the clouds and how they were still moving despite the chaos on the ground. This allowed me to truly appreciate the small things and the beauty of everyday life.” – 2nd year, Johannesburg, South Africa.

“In an attempt to salvage my life, I decided to fix my sleep and wake up before dawn […] Surprisingly, in those silent and peaceful hours I realized how blessed I was to be able to see the beauty of this earth. Watching the sunrise and witnessing nature come to life helped me unravel the chaos inside me and gave me hope.” – 5th year, Riyadh, Saudi Arabia.

## Discussion

This study suggested that the pandemic acted as a disorientating dilemma for medical students leading them to consider their own professional identity and the type of doctor they wanted to become. Students became more aware of the social and global role of a doctor in addressing health inequities. Students felt part of a wider healthcare community and showed greater appreciation towards person-centred care. Furthermore, students became more aware of their personal needs and priorities, and the importance of self-care.

More specifically, students realised the complexity of their emerging roles as future physicians, such as their role to protect and advocate for vulnerable and disadvantaged groups. Similar to Wu et al.’s findings [22], the pandemic caused students to reflect on the importance of leadership, advocacy, and the development of positive role models to inspire future generations. In accordance with Mezirow’s theory of transformational learning [10], through this process students had the dilemma to re-examine their pre-existing epistemic and sociocultural assumptions concerning the role of doctors, and to explore new, transformed perspectives of what it means to be a doctor. One could argue that without the presence of Covid-19 as such a sudden and potent disorientating dilemma, it is possible that students would not have developed these important insights at this early stage of their professional career. Furthermore, similar to previous studies [23, 24], working in the frontline during the pandemic led to higher emotional connection with other medical students, doctors and healthcare professionals, allowing medical students to feel a valuable member of a wider global community. This process has potentially enabled some students to adopt the values, dispositions, and aspirations of the healthcare community [7].

In response to the pandemic, students’ reflections suggested that they developed both emotion-focused coping strategies, such as emotional expression through engagement with the arts, as well as problem-solving coping strategies, such as volunteering. These mechanisms were used to gain a sense of agency during uncertain times. Both strategies may be indicators of higher psychological adjustment as a result of a disorientating dilemma. These findings echo Cheng’s et al. theory of ‘coping flexibility’ [25] who found that, although Covid-19 was an objectively uncontrollable stressor, resilient copers were able to identify and distinguish aspects that were still controllable, and act upon them to deal with the stressor. In this study, acting upon the issues that students could control, such as volunteering to work in the frontline to address the risk of Covid-19 on the society, not only allowed them to cope with a stressful situation, but also led to a higher sense of pride and achievement.

Nevertheless, the tension between protecting one’s wellbeing and making sacrifices for the benefit of the society needs to be acknowledged. Many students expressed their uncertainty as to whether they should prioritise their own needs and sense of wellbeing, or their sense of duty. Similar studies focusing on the impact of the pandemic found that medical students were not prepared to cope with this tension [2], which further increased the mental health challenges that students faced [3, 4]. As such, future PIF initiatives should focus on supporting students to cope with conflicting emotions and priorities. Stetson et al. [11] came to similar conclusions and highlighted the need for preparing students to balance their professional duties with their personal needs and values. Considering that the links between students’ wellbeing and PIF is currently underexplored, future research should focus on the impact of mental health and wellbeing support on developing professional identities. Longitudinal research would also be beneficial to understand the interplay between wellbeing and students’ PIF over time.

In this study we employed a unique way of capturing students’ perspectives. Engagement with the arts through the Global Creative Competition offered a more inclusive medium to access complex reflections, while simultaneously empowering students to safely express them. A similar study engaging medical students with creative enquiry during the pandemic also found that arts allowed students to engage with the complexity of what it is to be or become a doctor, and to work across hierarchies and disciplines to build connection and solidarity [26]. The General Medicine Council [12] has also suggested that arts-based reflective practice should be more widely included in medical trainings to foster wellbeing, resilience, self-awareness, and combat potential student burnout. Therefore, we suggest that the inclusion of arts and humanities in the medical curriculum and training, particularly where complex issues and situations are encountered, should be prioritised in future research, practice, and policy guidelines.

There are a number of implications of this study for medical educators world-wide. Firstly, the arts should be utilised more widely, as it serves as a powerful stimulus to support the development of medical students’ PIF. Medical students are increasingly interested in issues surrounding health inequalities particularly since the Covid-19 pandemic, and there should be opportunities within the curriculum to explore local and global inequalities. Furthermore, there should be opportunities for students to contribute meaningfully in addressing such inequalities through service-learning approaches or structured volunteering. Lastly, the development of professional identity is not always a straightforward path and often impacts on students' own wellbeing. It is therefore crucial that educators design into programmes formal support systems to give medical students the space and time to reflect on their emergent identities as a doctor.

### Strengths and limitations

Whilst the impact of the Covid-19 pandemic on medical student PIF has been previously researched in single medical schools or countries, this study draws upon reflections from 155 medical students from 28 countries. Despite varying contexts, many similar themes were identified in how students responded to the pandemic and how this impacted on their PIF. The analysis of the reflections was also undertaken by three members of the authorship team, and themes and codes were discussed with the wider team to ensure that we were cognisant of our own assumptions about the interpretations of students’ reflections.

However, the findings represent only a snapshot in time and thus students' thoughts and reflections about their professional identity and response to the pandemic will likely evolve over time. The exclusive online access to the competition and the lack of inter-rater reliability calculations also present limitations. Furthermore, students were writing their reflections for a competition in English, with a limited word count and a specific brief to respond to. The next iteration of the Global Competition addressed the former, with students being able to submit entries in their preferred language. These factors may have impacted on what students chose to write about, how they presented themselves through their reflections, and potentially hindered richer and deeper thoughts.

## Conclusions

The pandemic as a disorientating dilemma acted as a transformative experience for students around the world, in terms of their own professional identity formation and considering the type of doctor that they wanted to become. Medical educators should recognise this and provide space and opportunities for students to reflect on and discuss their ongoing identity formation as students encounter other disorientating dilemmas in their clinical training. The inclusion of arts and humanities within the medical curriculum and training is strongly recommended to provide an avenue for students to access and express complex emotions and experiences.

Many students reflected on their desire to adopt a more socially accountable and community engaged approach to their work as a future doctor. Further consideration should be given to how community engagement might be further integrated into medical curricula and how this might influence and support students' PIF.

The pandemic had a strong impact on students' mental health and wellbeing as a result of volunteering in clinical contexts, isolation and loneliness, or anxiety about their own and others’ health. Medical schools need to recognise this and provide ongoing resources and support for these students. Students’ mental health and wellbeing is fundamental to their progression and transition to becoming a doctor, and further work is therefore needed to better understand the links between mental health, wellbeing and professional identity frameworks, and the implications of this for the role of the future doctor.

## Data Availability

The datasets used and/or analysed during the current study are available from the corresponding author on reasonable request.
